# The prevalence of dental erosion and associated risk factors in 12-13-year-old school children in Southern China

**DOI:** 10.1186/1471-2458-10-478

**Published:** 2010-08-12

**Authors:** Ping Wang, Huan Cai Lin, Jian Hong Chen, Huan You Liang

**Affiliations:** 1 Department of Stomatology, the Third Affiliated Hospital, Sun Yat-sen University, Guangzhou, Guangdong, China; 2 Department of Dental Public Health, Guanghua School of Stomatology, Sun Yat-sen University, Guangzhou, Guangdong, China

## Abstract

**Background:**

Dental erosion has been investigated in developed and developing countries and the prevalence varies considerably in different countries, geographic locations, and age groups. With the lifestyle of the Chinese people changing significantly over the decades, dental erosion has begun to receive more attention. However, the information about dental erosion in China is scarce. The purpose of this study was to explore the prevalence of dental erosion and associated risk factors in 12-13-year-old school children in Guangzhou, Southern China.

**Methods:**

This cross-sectional survey was performed by two trained, calibrated examiners. A stratified random sample of 12-13-year-old children (774 boys and 725 girls) from 10 schools was examined for dental erosion using the diagnostic criteria of Eccles and the index of O'Sullivan was applied to record the distribution, severity, and amount of the lesions. Data on the socio-economic status, health behaviours, and general health involved in the etiology of dental erosion were obtained from a self-completed questionnaire. The analyses were performed using SPSS software.

**Results:**

At least one tooth surface with signs of erosion was found in 416 children (27.3%). The most frequently affected teeth were the central incisors (upper central incisors, 16.3% and 15.9%; lower central incisors, 17.4% and 14.8%). The most frequently affected surface was the incisal or occlusal edge (43.2%). The loss of enamel contour was present in 54.6% of the tooth surfaces with erosion. Of the affected tooth surfaces, 69.3% had greater than one-half of the tooth surface was affected. The results from logistic regression analysis demonstrated that the children who were female, consumed carbonated drinks once a week or more, and those whose mothers were educated to the primary level tended to have more dental erosion.

**Conclusions:**

Dental erosion in 12-13-year-old Chinese school children is becoming a significant problem. A strategy of offering preventive care, including more campaigns promoting a healthier lifestyle for those at risk of dental erosion should be conducted in Chinese children and their parents.

## Background

Dental erosion is defined as the loss of hard dental tissue due to the chemical influence of extrinsic and intrinsic acids without bacterial involvement [1]. It is becoming an increasingly important factor when considering the long-term health of the dentition. If dental erosion is not controlled and stabilized, the child may suffer from severe tooth surface loss, tooth sensitivity, over closure, poor aesthetics, or even dental abscesses in the affected teeth [2]. Erosive lesions frequently require preventive and restorative treatments, which will add the family and government's public health burden. The etiology of dental erosion is multi-factorial and not fully understood. Currently, the increased consumption of acidic foods and carbonated beverages is becoming an important factor in the development of dental erosion [3]. The acidic attack leads to an irreversible loss of dental hard tissue, which is accompanied by a progressive softening of the surface [3].

Epidemiologic surveys have investigated dental erosion in developed and developing countries. These results have shown that the prevalence of dental erosion varies considerably in different countries, geographic locations, and age groups (Table 1). With the rapid economic development in China, the lifestyle of the Chinese people, including diet and attitude toward dental health, has changed significantly through the decades. Dental disorders, such as dental erosion, have begun to receive more attention, but the information about dental erosion is scarce.

**Table 1:** Prevalence of dental erosion in 11-14-year-old children

Author	Year	Country	Age	Sample size	Present (%)	Exposed Dentine (%)	Teeth examined
Al-Dlaigan [29]	2001	UK	14	418	100	52	All permanent teeth
Deery [13]	2000	UK	11-13	125	37	0	Upper permanent incisors
		USA	11-13	129	41	0	Upper permanent incisors
Ganss [30]	2001	German	11.4	1000	11.6	0.2	All permanent teeth
Al-Majed [8]	2002	Saudi Arabia	12-14	862	95	26	Upper permanent incisors and first molars
Dugmore [21]	2004	UK	12	1753	59.7	2.7	permanent incisors and first molars
Peres [19]	2005	Brazil	12	499	13.0	0.32	Upper permanent incisors
Caglar [10]	2005	Turkey	11	153	28	0	All permanent teeth
EL Karim [14]	2007	Sudan	12-14	157	66.9	0	Upper permanent incisors
Auad [12]	2007	Brazil	13-14	458	34.1	0	All permanent teeth
Waterhouse [31]	2008	Brazil	13-14	458	34.1	0	All permanent teeth
Talebi [24]	2009	Iran	12	483	38.1	4.0	Upper permanent incisors
Correr [11]	2009	Brazil	12	389	26	35	All permanent teeth

The sample age in studies of the prevalence of dental erosion was 3-50 years old [4]. Many studies have focused on 12-year-old children (Table 1) because the permanent incisors and first molars of children at this age have been exposed to potential etiologic factors in the mouth for a considerable duration compared to other teeth. Furthermore, the differentiation of erosion from attrition or abrasion is easier at this age compared to adults.

The objective of the present study was to evaluate the dental erosion status among 12-13-year-old school children in Guangzhou, Southern China, including the prevalence, distribution, and severity of dental erosion in the permanent dentition at the tooth and surface level. This investigation also aimed to explore the associated socioeconomic and behavioral risk factors influencing the prevalence of dental erosion.

## Methods

### Sample

Guangzhou is the capital city of the Guangdong province, which is located in Southern China. The climate is hot and long in the summer due to its location in the southern subtropical zone. The summer is 6 months in length (May to October). Guangzhou is a rapidly developing city with a population of approximately 10 million. The gross domestic product (GDP) per capita was USD 9,302 in 2007, and Guangzhou ranked as the third economically developed city in China, following Beijing and Shanghai [5].

A cross-sectional study was conducted in Guangzhou by a stratified multi-stage cluster sampling. A pilot study was performed before the formal study. The prevalence of dental erosion was 20.8% for 175 school children (12-13 years of age) examined from an urban junior high school. A total of 1,500 school children were proposed in our study according to the sample size formula for the estimation of prevalence (α = 0.05) in order to decrease study error. Guangzhou is comprised of 10 urban districts and 2 suburban districts. The population ratio between the urban and suburban regions was approximately 4:1 [5]. Four urban districts and one suburban district were selected by simple random sampling. A list of all junior high schools in these districts was obtained from the local Department of Education. Two junior high schools were selected by simple random sampling in each district. In each school, three classes of grade one students were selected by the same sampling method and 140-160 children were cluster-selected. All of the selected children were 12-13 years of age. Those who were with orthodontic appliances, enamel defect accompanied by a loss of tooth substance, and fractured or missing teeth of the incisors or the first molars were excluded. A total of 1,499 children (774 boys and 725 girls), 12-13 years of age, from 10 junior high schools were invited to participate in the study.

The study protocol was approved by the Research Ethics Committee of Guanghua School of Stomatology of Sun Yat-sen University. An informed consent letter regarding the aim and importance of the study was signed by the children and the parents/guardians before starting the survey, which assured that children participated in the study on their own accord.

### Calibration of examiners

Training and calibration exercises were conducted prior to the study. An experienced dental epidemiologist who had a high level of education and experience, was responsible for training and calibrating two examiners. The diagnostic criteria of dental erosion were thoroughly discussed with the examiners. A range of dental erosion levels based on the diagnosis via photographic images was reviewed in the calibration exercise. The epidemiologist also provided instruction for the examiners during the pilot study.

To assess the reproducibility of the diagnostic criteria, approximately 10% of the subjects were re-examined (only the labial surfaces of the upper incisors). In every dental examination section at each school, approximately 15 students in intervals of 10 were re-examined by the same examiner and another examiner. The results of the duplicate examinations were used to estimate the intra- and inter-examiner reliability.

### Clinical Examination

The two previously calibrated examiners participated in the clinical examinations and visited the selected schools. The clinical examinations were performed in well-lit classrooms or in shaded places under natural light using plane mouth mirrors and sterilized cotton to remove debris. The central incisors, lateral incisors, and first molars in the upper and lower jaws were examined. The diagnostic criteria of dental erosion proposed by Eccles [6] were used in this study. The index of O'Sullivan [7] was adopted to record the distribution, severity, and amount of affected teeth. This index is especially designed for epidemiologic surveys and for the diagnosis of erosion in children to determine treatment options.

O'sullivan index for measurement of dental erosion:

Site on erosion on each tooth

Code A: Labial or buccal only

Code B: Lingual or palatal only

Code C: Occlusal or incisal only

Code D: Labial and incisal/occlusal

Code E: Lingual and incisal/occlusal

Code F: Multi-surface

Grade of severity (worst score for an individual tooth recorded)

Code 0: Normal enamel

Code 1: Matt appearance of the enamel surface with no loss of contour

Code 2: Loss of enamel only (loss of surface contour)

Code 3: Loss of enamel with exposure of dentine (enamel-dentin junction visible)

Code 4: Loss of enamel and dentine beyond enamel-dentin junction

Code 5: Loss of enamel and dentine with exposure of the pulp

Code 9: Unable to assess (e.g. tooth crowned or large restoration)

Area of surface affected by erosion

Code -: Less than half of surface affected

Code +: More than half of surface affected

### Questionnaire

The school children completed a questionnaire at the schools prior to the clinical examination. The questionnaire was designed to reflect the socio-economic status, behavioural factors, and general health involved in the etiology of erosion, as proposed by Lussi [3] and in other studies [8,9]. The pilot study was carried out to test and refine the questionnaire.

The questionnaire included questions about general information (gender and age), socio-economic status, occupation and education levels of the parents, oral hygiene habits, frequencies of ingesting certain beverage types, amount of acidic drink intake per week (including carbonated drinks, sport drinks, lemon tea, and fruit juices), special drinking habits, general health (including frequency of vomiting and heartburn or nausea in this study), and vitamin C supplements. The frequency of swimming in summer was also included in the questionnaire because of possible lower pH value in the water of swimming pools [3].

### Statistical analyses

The study data were entered into a computer using Epidata (version 3.0) and analyzed using SPSS software (version 13.0). Intra- and inter-examiner agreement was evaluated using Cohen's kappa. Descriptive analysis was conducted to describe the prevalence and characteristics of dental erosion. A two-step approach was used to analyze risk factors of dental erosion. First, bivariate analysis was used to test the relationship between dental erosion and the associated factors. Then, a logistic regression analysis was used to analyse the factors that were independently related to the presence of erosion. The variables (*P *< 0.5) in the bivariate analyses were entered into a logistic regression model in a forward fashion. The level of statistical significance was set at 5%.

## Results

### Clinical data

**Figure 1:**
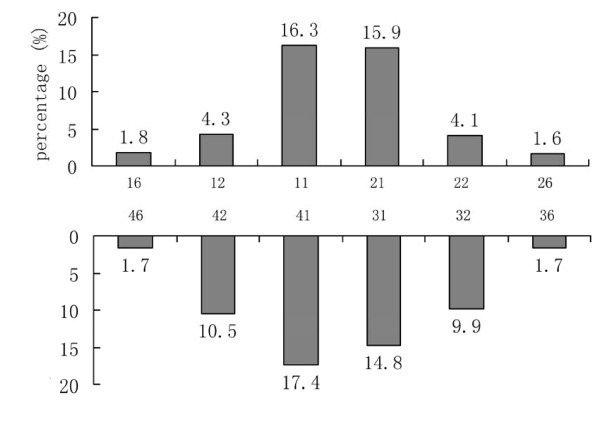
Percentage of teeth affected with any signs of dental erosion for children aged 12-13 years

In our study, 1,499 school children (774 boys and 725 girls), 12-13 years of age, were examined. Signs of erosion on at least one tooth surface occurred in 416 children and the prevalence of dental erosion was 27.3% (95% CI = 25.0%-29.6%). The prevalence of 12- and 13-year-old children was 25.5% and 29.0%, respectively (*P *= 0.151). There was no significant difference in the prevalence of dental erosion in children in urban and suburban areas (26.2% *vs. *28.9%; *P *= 0.087). The most frequently affected teeth were the central incisors, followed by the lateral incisors and first molars (Figure 1).

A total of 17,988 teeth (53,964 tooth surfaces) were examined in this study and 1,895 teeth (3,288 surfaces) showed signs of erosion. The most frequently affected surface was the incisal/occlusal edge (43.2%), followed by the labial and incisal/occlusal surfaces (22.7%). Regarding the severity of dental erosion, most affected tooth surfaces exhibited the loss of enamel contour (54.6%). Of the affected tooth surfaces, 69.3% showed that greater than one-half of the tooth surface was affected (Table 2).

**Table 2:** Percentage of affected tooth surfaces, severity, and area of the surfaces affected by dental erosion according to the number of teeth/surfaces

	No. of teeth/surfaces	Percentage (%)
Affected surface		
Labial or buccal only	146	7.7
Lingual or palatal only	72	3.8
Incisal or occlusal only	818	43.2
Labial and incisal/occlusal	430	22.7
Lingual and incisal/occlusal	58	3.0
Multi-surface	371	19.6
Total (teeth)	1895	100.0
Severity		
Matt appearance of the enamel	1447	44.0
Loss of enamel only	1795	54.6
Loss of enamel with exposure of dentin (enamel-dentin junction visible)	46	1.4
Total (surfaces)	3288	100.0
Area affected		
Less than half of surface affected	1008	30.7
More than half of surface affected	2280	69.3
Total (surfaces)	3288	100.0

Diagnostic reproducibility was assessed by examining the labial surfaces of upper incisors. Duplicate examinations of a total of 150 students yielded two kappa values (both were 0.92) for intra-examiner reproducibility and a kappa value of 0.73 for inter-examiner reproducibility, which indicated good agreement for reproducibility of erosion.

### Questionnaire data/associated risk factors

Socio-economic factors were based on the occupation and education levels of the parents because the income of the children being examined was unknown. The results analysed with a chi-square test indicated that children with mothers that had higher levels of education tended to have a lower prevalence of dental erosion (*P *< 0.05; Table 3). There was a significant difference in the prevalence of dental erosion in relation to the frequency of consumption of carbonated drinks (*P *< 0.05; Table 4). No significant association was found between dental erosion and oral hygiene habits, general health, and vitamin C supplements (Table 5).

**Table 3:** Socio-economic factors based on the children's gender, parent's level of education, and occupation in relation to the prevalence of dental erosion

Variables	Total No. of children	Dental erosion		
		
		No. of children	%	P
Gender				
Male	774	199	25.7	0.068
Female	725	217	29.9	
Father's education				
Primary	544	169	31.1	0.069
Secondary	633	169	26.7	
College and postgraduate	322	78	24.2	
Mother's education*				
Primary	665	196	29.5	0.035
Secondary	575	165	28.7	
College and postgraduate	259	55	21.2	
Father's occupation				
Employers/professional	327	93	28.4	0.909
Employees/non-professional	857	238	27.8	
Unemployed	315	85	27.0	
Mother's occupation				
Employers/professional	244	59	24.2	0.384
Employees/non-professional	948	268	28.3	
Unemployed	307	89	29.0	

*P < 0.05, Chi-squared test.

**Table 4:** The frequency of consumption of drinks and foods, and special drinking habits in relation to the prevalence of dental erosion

Variables	Total No. of children	Dental erosion		
		
		No. of children	%	*P*
Frequency of carbonated drinks*				
< once a week	936	243	26.0	0.046
≥once a week	563	173	30.7	
Frequency of sport drinks				
< once a week	1089	305	28.0	0.719
≥once a week	410	111	27.1	
Frequency of lemon tea				
< once a week	1102	310	28.1	0.585
≥once a week	397	106	26.7	
Frequency of succade				
< once a week	1160	314	27.1	0.275
≥once a week	339	102	30.1	
Frequency of fruits				
< once a week	180	52	28.9	0.716
≥once a week	1319	364	27.6	
Frequency of fruit juices				
< once a week	940	253	26.9	0.348
≥once a week	559	163	29.2	
Frequency of chewing gum				
< once a week	817	233	28.5	0.468
≥once a week	682	183	26.8	
Amount of acidic drinks intake				
< 250 ml/week	408	103	25.2	0.406
250-1000 ml/week	962	275	28.5	
> 1000 ml/week	129	38	29.4	
Drinking with straw or not				
Drinking with straw	475	138	29.2	0.444
Drinking without straw	1024	278	27.1	
Method of drinking				
Sucking or holding drinks in mouth	328	81	24.7	0.162
Drinking straight away	1171	335	28.6	
Frequency of drinks taken at night				
Once or more monthly	82	26	31.7	0.418
Never/occasionally	1417	390	27.5	

*P < 0.05, Chi-square test

**Table 5:** Oral hygiene habits, general health, vitamin C supplements, and frequency of swimming in relation to the prevalence of dental erosion

Variables	Total No. of children	Dental erosion		
			
		No. of children	%	*P*
Frequency of brushing				
Once or less daily	459	136	29.6	0.281
Twice or more daily	1040	280	26.9	
Duration of brushing				
≤1 min	266	73	27.4	0.908
2 min	702	192	27.4	
≥3 min	531	151	28.4	
Types of toothpaste				
Fluoride	656	170	25.9	0.333
Non-fluoride	74	20	27.0	
Not sure	769	226	29.4	
Vomiting				
Once or more monthly	16	2	12.5	0.171
Never/occasionally	1483	414	27.9	
Heartburn or nausea				
Once or more monthly	9	0	-	0.136
Never/occasionally	1490	416	27.9	
Vitamin C supplements				
Yes	273	78	28.6	0.749
No	1226	338	27.6	
Frequency of swimming in summer				
Once or more weekly	732	193	26.4	0.229
Never/occasionally	767	223	29.1	

Chi-square test

When other variables were taken into account, factors that remained statistically significant were gender, frequency of consumption of carbonated drinks, and the level of education of the mother (Table 6). The children who were female, consumed carbonate drinks more than once a week, and had mothers that were educated to the primary level had more dental erosion (*P *< 0.05).

**Table 6:** Logistic regression analysis results in relation between the prevalence of dental erosion and children's gender, frequency of consumption of carbonated drinks, and mother's level of education

					95% CI for OR	
					
Variables	B	SE	P	OR	lower	upper
Gender						
Male*						
Female	0.247	0.117	0.035	1.281	1.023	1.624
Carbonated drinks						
< Once a week*						
≥Once a week	0.262	0.120	0.029	1.299	1.028	1.643
Mother's education						
Primary*						
Secondary	-0.043	0.126	0.734	0.958	0.749	1.266
College and postgraduate	-0.431	0.175	0.013	0.650	0.461	0.914
Constant	-1.358	0.204	< 0.001	0.257		

B: regression coefficient; SE: standard error; P: significance; OR: odds ratio

*parameters

Omnibus Tests of Model Coefficients: X^2 ^= 22.105, df = 4, P < 0.001

Further analyses were done for the intrinsic and extrinsic acid contact by gender. There was no significant difference in the vomiting, heartburn or nausea, frequency of consumption of carbonated drinks, lemon tea, fruit juices and chewing gum between the genders (*P *= 0.382, 0.813, 0.613, 0.908, 0.801, and 0.767, respectively). Girls had a higher frequency of consumption of fruits and succade than boys (*P *< 0.001 and *P *= 0.014, respectively), while boys drank more sport drinks than girls (*P *< 0.001). Girls liked to suck or hold drinks in their mouths (*P *= 0.045); however, boys consumed a greater amount of acidic drinks than girls (*P *< 0.001).

## Discussion

The prevalence of dental erosion of 27.3% in the current study was similar to the results of Caglar et al. [10] and Correr et al. [11], which indicated that 28% of 11-year-old children and 26% of 12-year-old children had permanent teeth affected by erosion, respectively. However, the prevalence ranging from 11.6%-100% was determined during recent surveys on the permanent dentition of children in different countries (Table 1). The variation in prevalence among these studies may be partially explained by differences in the diagnostic criteria and indices. Furthermore, socio-economic, cultural, and geographic factors could influence the outcome of prevalence data.

The central incisors in the upper and lower jaws were affected predominantly, followed by the lateral incisors. The first molars in the upper and lower jaws were less affected by erosion. The upper central incisors of children were usually reported to be frequently affected by erosion [12-14]. In our study, the lower central incisors were affected predominantly as well. Both the upper and lower incisors are located in the front of the oral cavity, which predisposes these teeth to erosion by extrinsic acids, such as acidic beverages. Moreover, the lower central incisors are the first erupting teeth, which are exposed to erosive challenges for a longer period of time. These may be the possible reasons for this finding.

The most frequently affected surface was the incisal or occlusal only (43.2%) for all teeth, followed by the labial and incisal/occlusal surfaces. Al-Majed et al. [8] also determined that 91% of dental erosion occurred on the incisal/occlusal surfaces of 12-14-year-old Saudi boys. The results of other surveys varied. For example, Auad et al. [12] and Mangueira et al. [15] reported that the palatal surfaces were the most affected. The consumption of erosive drinks and food was also shown to be strongly associated with erosion on the facial and occlusal surfaces, while severe palatal erosion occurred infrequently and were highly associated with chronic vomiting [16]. The predominant affected surfaces of erosion on the incisal/occlusal might result from these tooth surfaces being predisposed to physical impacts from mastication, which promotes the effect of erosion. Although distinguishing erosion from abrasion/attrition in the incisal/occlusal surfaces at the late stages of primary and permanent dentition is difficult, the dentition of 12-13-year-old children is during the early stages and the appearance of the incisal/occlusal surfaces as grooving/cupping is mainly due to erosion [17]. According to the differential diagnosis of dental erosion proposed by Gandara et al. [18], incisal surface contour appearing flat and shiny was defined as abrasion or attrition and was not included in the records in our study.

The severity of erosive lesions in our study demonstrated that the loss of enamel contour occurred most frequently (54.6%), and only a small proportion of tooth surfaces were affected with dentine exposure (1.4%). Several surveys found that all affected children of their study samples exhibited erosion with no exposed dentin. Peres et al. [19] reported that enamel loss was the most prevalent type of dental erosion for 12-year-old school children in Brazil. However, Al-Majed et al. [8] examined 862 Saudi Arabian boys 12-14 years of age, and 26% of these children exhibited pronounced dental erosion (erosion into the dentin or pulp). The frequent ingestion of carbonated soft drinks at nighttime could contribute to the high percentage of pronounced dental erosion in these Saudi boys.

Associations between dental erosion and the variables under study were investigated through processes of bivariate and multivariate analyses. A logistic regression analysis contributed to eliminate confounding factors that may mask an actual association or falsely demonstrate an apparent association between the study variables. The results of logistic regression analysis demonstrated that the gender of the children, frequency of consumption of carbonated drinks, and the level of education of the mothers were independent risk factors for dental erosion in school children of Southern China.

A significantly higher prevalence of dental erosion was found in girls (29.9%) than in boys (25.7%), which is in agreement with the results of previous investigation of 12-year-old Cuban children by Künzel et al. [20]. However, several surveys had found a significantly higher prevalence in boys than in girls [21,22], while Correr et al. [11] and Peres et al. [19] found no difference between the genders. In the present study, there was no significant difference in the frequency of consumption of carbonated drinks between the genders. Girls ate more succade and fruit, and were more likely to suck drinks than boys, but these variables had no significant difference between children with and without dental erosion. These results indicate that the reason of gender difference in the occurrence of dental erosion was not clear and further studies are necessary on this issue.

In the literature, the influence of socio-economic status is somewhat conflicting [9,23]. Children whose mothers had higher levels of education exhibited fewer lesions, which is in agreement with the study by Harding et al. [23]. Some studies reported no relationship between erosion and social class based on the occupation of the father and education of the mother [9]. In this study, the relationship between the level of education of the mother and the presence of erosion in the children may be due to the influence of the level of education on the lifestyle of the family. Mothers who had higher levels of education may have more knowledge of oral hygiene and better oral health habits.

The frequency of consumption of carbonated drinks was significantly related to dental erosion in the present study, which supported the results of previous surveys [24-26]. Daily fluid consumption is high due to the hotter climate in Guangzhou, particularly the consumption of carbonated soft drinks, which are becoming increasingly available at a reasonable cost and are popular due to their taste. Outputs of carbonated drinks in the Guangdong province increased to approximately 2 billion litres (22.7% of the total output in China) from January - October 2007, and ranked first of all 31 provinces/municipalities [27]. A recent study performed in seven cities of China, including Guangzhou, reported that carbonated drinks were still the predominant beverage preferred by school children. The frequency of consumption of carbonated drinks has increased greatly compared to 8 years ago [28]. There is a need for intervention programs aimed at the increasing consumption of soft drinks among school children in China, such as a media campaign, classroom workshops, school meal modification, and parental support.

The present study had limitations because of recall bias, the cross-sectional study design, and the relatively small sample size of suburban residents. Standardization of the indices and the teeth examined would facilitate the comparisons of different studies. More research should be done to determine the differences in tooth erosion and the main risk factors between urban and rural children. The prospective epidemiologic design will be of benefit for elucidation of the risk factors of the disease.

## Conclusions

Our study provides evidence that dental erosion is becoming a significant problem in Chinese school children. Dental erosion should receive more attention that promotes awareness in dentists to make an early diagnosis and to evaluate the different etiologic factors that identify children at risk in China. Children who were female, consumed carbonated drinks once a week or more, and had mothers that were educated to the primary level tended to have more dental erosion. A strategy of offering preventive care, including more campaigns promoting a healthier lifestyle for those at risk of dental erosion, should be conducted for school children and their parents in China in order to reduce the medical burden of the government and families.

## Competing interests

The authors declare that they have no competing interests.

## Authors' contributions

**PW**: design of the study, performing the dental examination, data management and analysis, writing of the manuscript. **HCL**: design of the study, training and supervising fieldworkers, revised the manuscript critically for important intellectual content. **JHC**: design of the study, project co-ordination. **HYL**: design of the study, implementation and supervision of field work. All authors read and approved the final manuscript.

## Pre-publication history

The pre-publication history for this paper can be accessed here:

http://www.biomedcentral.com/1471-2458/10/478/prepub
